# A Polycarbonate-Assisted Transfer Method for van der Waals Contacts to Magnetic Two-Dimensional Materials

**DOI:** 10.3390/mi15111401

**Published:** 2024-11-20

**Authors:** Kunlin Yang, Guorui Zhao, Yibin Zhao, Jie Xiao, Le Wang, Jiaqi Liu, Wenqing Song, Qing Lan, Tuoyu Zhao, Hai Huang, Jia-Wei Mei, Wu Shi

**Affiliations:** 1 State Key Laboratory of Surface Physics and Institute for Nanoelectronic Devices and Quantum Computing, Fudan University, Shanghai 200433, China; klyang21@m.fudan.edu.cn (K.Y.); grzhao22@m.fudan.edu.cn (G.Z.); ybzhao21@m.fudan.edu.cn (Y.Z.); wqsong22@m.fudan.edu.cn (W.S.); 22110190025@m.fudan.edu.cn (Q.L.); tyzhao23@m.fudan.edu.cn (T.Z.); 2 Zhangjiang Fudan International Innovation Center, Fudan University, Shanghai 201210, China; 3 Department of Physics, Southern University of Science and Technology, Shenzhen 518055, China; 12332948@sustech.edu.cn (J.X.); wangl36@sustech.edu.cn (L.W.); 23210190022@m.fudan.edu.cn (J.L.); meijw@sustech.edu.cn (J.-W.M.); 4 Shanghai Frontiers Science Research Base of Intelligent Optoelectronic and Perception, Institute of Optoelectronic and Department of Material Science, Fudan University, Shanghai 200433, China; huangh@fudan.edu.cn

**Keywords:** van der Waals contacts, dry-transfer method, magnetic 2D materials, spintronics

## Abstract

Magnetic two-dimensional (2D) materials have garnered significant attention for their potential to revolutionize 2D spintronics due to their unique magnetic properties. However, their air-sensitivity and highly insulating nature of the magnetic semiconductors present substantial challenges for device fabrication with effective contacts. In this study, we introduce a polycarbonate (PC)-assisted transfer method that effectively forms van der Waals (vdW) contacts with 2D materials, streamlining the fabrication process without the need for additional lithography. This method is particularly advantageous for air-sensitive magnetic materials, as demonstrated in Fe_3_GeTe_2_. It also ensures excellent interface contact quality and preserves the intrinsic magnetic properties in magnetic semiconductors like CrSBr. Remarkably, this method achieves a contact resistance four orders of magnitude lower than that achieved with traditional thermally evaporated electrodes in thin-layer CrSBr devices and enables the observation of sharp magnetic transitions similar to those observed with graphene vdW contacts. Compatible with standard dry-transfer processes and scalable to large wafer sizes, our approach provides a straightforward and effective solution for developing complex magnetic heterojunction devices and expanding the applications of magnetic 2D materials.

## 1. Introduction

Two-dimensional (2D) materials have captured significant attention due to their unique properties and potential in cutting-edge fields such as nanoelectronics, spintronics, and opto-electronics [1,2]. Among these materials, magnetic 2D materials are especially noteworthy for their intrinsic magnetic properties and critical role in advancing spintronics [3,4]. For instance, bilayer antiferromagnetic CrI_3_ has enabled new spintronic device concepts such as gate-tunable magnetic tunnel junctions and spin tunnel FETs [5]. Additionally, the magnetic semiconductor CrSBr has shown considerable promise in emerging fields like magneto-optics and antiferromagnetic spintronics, as highlighted in recent studies [6,7,8,9]. However, these materials often pose significant challenges; many are air-sensitive and the semiconducting magnetic 2D materials, crucial for developing gate-tunable spintronic devices, are highly insulating, leading to severe contact issues. This has prompted a focus on vertically stacked structures [10] and underscores the need for innovative contact methods to fully exploit their potential in spintronic devices with lateral configurations.

The ultra-thin nature of magnetic 2D materials makes them particularly susceptible to damage during traditional contact fabrication methods such as thermal or electron-beam (e-beam) evaporation [11,12]. These methods can introduce defects, disorder, and metal diffusion, thereby compromising the intrinsic magnetic textures of these materials and obstructing the accurate detection of their magnetic properties through transport measurements. Recent strategies, such as the physical transfer of pre-deposited metal electrodes, have been explored to form van der Waals (vdW) contacts, effectively mitigating issues like surface damage and Fermi-level pinning [13,14,15,16,17]. For example, Duan et al. successfully achieved an electron mobility of 260 cm^2^/V·s and a hole mobility of 175 cm^2^/V·s (two-terminal) at room temperature by transferring pre-fabricated metal electrodes with suitable work functions onto few-layer MoS_2_ using polymethyl methacrylate (PMMA); their result verifies the fundamental limits of an ideal metal–semiconductor interface [13]. However, these methods often involve complex preparatory steps, such as hexamethyldisilazane (HMDS) treatment to functionalize the SiO_2_/Si substrate in PMMA-assisted transfers. Moreover, while these techniques have been extensively applied to conventional 2D materials like graphene and transition metal dichalcogenides [13,14,17], their application to magnetic 2D materials remains limited, restricting their practical adoption.

In this work, we introduce a straightforward polycarbonate (PC)-assisted transfer method for van der Waals contacts to 2D materials, specifically tested for various magnetic 2D materials. This method not only simplifies the nanodevice fabrication process without additional lithography requirements, ideal for air-sensitive materials like Fe_3_GeTe_2_, but also achieves optimal contacts in vdW material semiconductors such as CrSBr, which exhibit contact resistances four orders of magnitude lower compared to those with traditional thermally evaporated electrodes. Magneto-transport measurements show clear sharp antiferromagnetic-to-ferromagnetic transitions in bilayer CrSBr devices with transferred gold (Au) electrodes as well as with graphene electrodes, except for the devices with conventional thermally evaporated electrodes. Our approach thus preserves the delicate magnetic properties of these magnetic 2D materials and minimizes contact resistance significantly, enabling the exploration of their full potential in spintronic device applications.

## 2. Methods

### 2.1. Device Fabrication by Using the PC-Assisted Transfer Method

**Figure 1:**
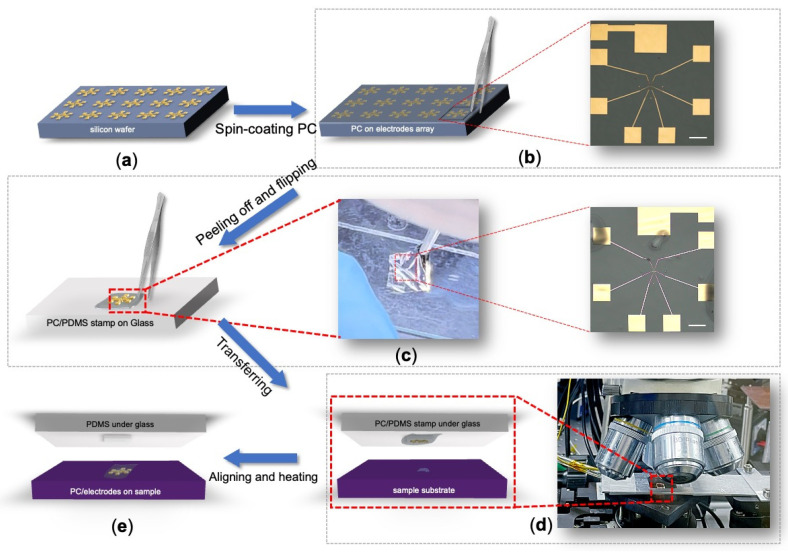
Schematic diagram of the PC-assisted transfer method for vdW contacts. Blue arrows indicate the sequence of operations, and red dashed lines highlight the magnified areas. (**a**) Au electrode arrays fabricated on a silicon wafer using thermal evaporation after lithography. (**b**) A silicon wafer spin-coated with a PC film, where the black rectangular frame indicates the area cut with a blade, representing the portion to be used. The right panel shows an optical image under a microscope, with a scale bar of 200 μm. (**c**) Illustration of the process where the PC film with electrodes is flipped and placed onto a PDMS stamp. Left panel: schematic of the operation. The middle optical image shows the Au electrodes transferred onto the PC film during the process, with a scale bar of 200 μm. Right panel: an image of the electrodes/PC on the PDMS stamp, with bubbles near the large electrode pads that will be removed during the heated transfer. Scale bar: 200 μm. (**d**) Alignment process. The target flake of 2D materials on a substrate is precisely aligned with the electrodes on the PDMS stamp and carefully stacked together using an XYZR transfer stage. Right panel shows a photograph of the motorized transfer stage inside a glovebox. (**e**) The transferred electrodes establish van der Waals contact with the sample. The PC film is melted when heating the sample to 180 °C and then dissolved in chloroform to complete the transfer.

The detailed procedure of our PC-assisted transfer method is meticulously outlined in Figure 1. Initially, an array of pre-designed Au electrodes is fabricated on a silicon (Si) substrate using either standard electron-beam lithography (EBL) or photolithography, followed by the thermal evaporation of a 50 nm-thick Au film (Figure 1a). After lithography and lift-off, the electrode array is immediately spin-coated with a polycarbonate (PC) solution, prepared by dissolving PC from Sigma-Aldrich (St. Louis, MO, USA) in chloroform at a 5% concentration. This solution is stirred overnight using a magnetic stirrer and then spin-coated onto the silicon wafer at 600 rpm for 6 s and subsequently at 4000 rpm for 1 min. The coated wafer is left to air-dry naturally overnight.

Once dry, the PC film with electrodes is carefully handled to ensure precision and avoid damage. Using a blade, the required portion of the PC film is cut from the substrate, and tweezers are used to lift one corner for easy handling (Figure 1b). A polydimethylsiloxane (PDMS) stamp of suitable size, prepared by mixing SYLGARD 184 (from DOW Silicones Deutschland GmbH, Wiesbaden, Germany) base polymer with curing agent in a 10:1 ratio and cured at room temperature for 24 h to allow bubbles to dissipate, is then placed on a clean glass slide to support the PC film. The PC film, now bearing the electrodes, is gently flipped onto the PDMS stamp ensuring it lies flat and is free from wrinkles (Figure 1c), a step demonstrated in the Supplementary Video S1 Material which shows the careful flipping and placement of the PC/electrode film onto the PDMS stamp.

For the actual transfer, the target 2D material flake cleaved on a 285 nm SiO_2_/Si substrate is precisely aligned with the electrodes on the PDMS stamp under a microscope using an XYZR transfer stage (Figure 1d). The sample is then heated to 180 °C after alignment and stacking to melt the PC film, which is later dissolved in chloroform to finalize the transfer (Figure 1e). For air-sensitive materials like Fe_3_GeTe_2_, the samples were handled entirely in a nitrogen-filled glovebox (with an oxygen level below 0.1 ppm) throughout the whole PC-assisted transfer process to prevent any potential degradation from H_2_O/O_2_ exposure.

### 2.2. Device Fabrication by Using Conventional Lithography Method

For control experiments, we fabricated nanodevices of magnetic semiconductor CrSBr using two types of electrodes: conventional thermally evaporated Au electrodes and graphene electrodes. The Au electrodes were patterned using standard EBL and deposited by thermal evaporation. The graphene electrodes were created by transferring the CrSBr flake via a standard dry-transfer technique [18] to form vdW contact with the pre-cleaved graphene stripes. Subsequently, electrodes of 3 nm Cr/50 nm Au were fabricated by standard EBL and deposited using thermal evaporation to make contacts with graphene electrodes.

### 2.3. Transport Measurement

Transport measurements were conducted using either an Oxford 9 T system with a base temperature of 1.6 K, or in the variable temperature insert of a cryogenic 12 T superconducting magnet with a base temperature of 1.4 K. Magnetoresistance, anomalous Hall effect, and I–V curves were measured using three SR830 lock-in amplifiers and an SR570 preamplifier (from Stanford Research Systems, Sunnyvale, CA, USA). The reference frequency of the signal was set to 17.77 Hz. During the measurement of the Fe_3_GeTe_2_ device, a fixed 1 MΩ resistor was connected in series. The contact resistance was calculated using the formula (*R*_2T_ − *R*_4T_)/2, and the relative magnetoresistance was defined as (*R*(B) − *R*(0T))/*R*(0T). A slow magnetic-field sweep rate of 13.3 Oe per second was used for the magnetoresistance measurements for CrSBr devices. CrSBr single crystals were synthesized via the chemical vapor transport (CVT) method [6].

## 3. Results

**Figure 2:**
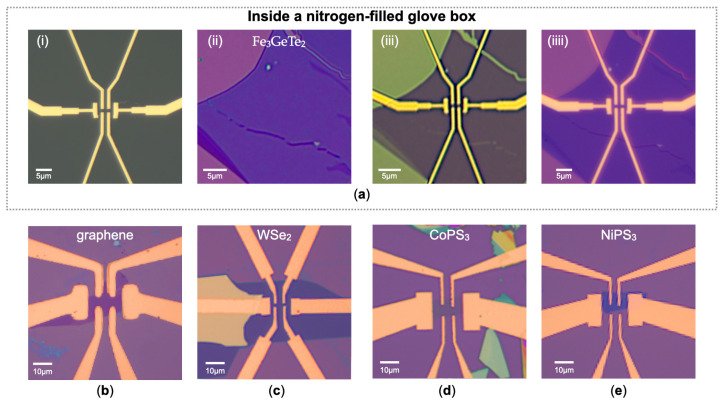
Transfer process in a glove box and optical images on different 2D materials. (**a**) (**i**–**iiii**) Sequential images showing the entire PC-assisted transfer process for establishing vdW contacts to air-sensitive ferromagnet Fe_3_GeTe_2_ inside the glovebox: (**i**) Optical image of the Au electrodes on a sacrificial silicon wafer layer, covered with spin-coated PC film. (**ii**) Cleaved few-layer Fe_3_GeTe_2_ sample on a SiO_2_/Si substrate. (**iii**) Optical image after the transfer process, showing the electrodes and the Fe_3_GeTe_2_ sample in van der Waals contact, with the melted PC film on top. (**iiii**) Optical image of the Fe_3_GeTe_2_ sample with transferred Au electrodes after removing the PC film in chloroform. (**b**–**e**) Optical images of devices with transferred Au electrodes for various 2D materials, including conventional 2D material graphene (**b**) and transition metal dichalcogenide WSe_2_ (**c**) as well as antiferromagnetic 2D materials CoPS_3_ (**d**) and NiPS_3_ (**e**). All scale bars are indicated in the images.

Our PC-assisted transfer method offers broad applicability across a diverse range of 2D materials for vdW contacts. This technique simplifies the transfer of metal electrodes, enabling precise alignment with sample flakes as small as a few microns. It accommodates the transfer of metal electrodes with any pre-designed patterns or in large-scale arrays. We present optical images of the electrodes and the air-sensitive ferromagnetic material Fe_3_GeTe_2_ before and after transfer in the nitrogen-filled glovebox in Figure 2a(i–iiii), where the shortest distance between adjacent electrodes is 1 µm. Figure 2a(iii) and Figure 2a(iiii) show the melted PC film on the sample and the material after the PC film is washed away in chloroform, respectively. We have successfully applied this method to fabricate vdW contacts to both conventional 2D materials like graphene and WSe_2_, as well as magnetic materials including CoPS_3_ and NiPS_3_, as demonstrated in Figure 2b–e. For conventional 2D materials such as graphene, using PC-assisted transfer Au electrodes, we achieved an ultra-low contact resistance less than 100 Ω from four-terminal measurements, comparable to those reported using the PMMA-assisted transfer method [13]. Although our method is also effective for semiconducting transition metal chalcogenides like MoS_2_, our main focus of this work has been on its application to magnetic 2D materials, where contact issues have not yet been thoroughly explored with transferred electrode techniques.

**Figure 3:**
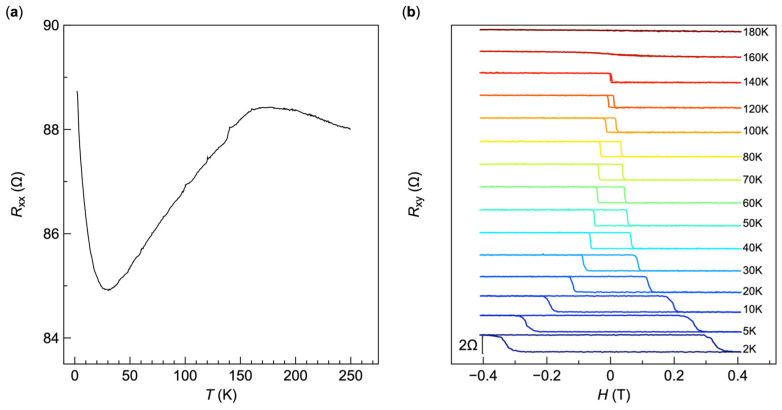
Electrical transport characterization of air-sensitive ferromagnet Fe_3_GeTe_2_ device with the PC-assisted transferred Au electrodes. (**a**) Four-terminal resistance Rxx versus temperature curve of the Fe_3_GeTe_2_ device, showing metallic behavior with good contact properties. The optical image of the device is shown in Figure 2e. (**b**) Hall resistance Rxy as a function of magnetic field measured at various temperatures from 2 K to 180 K (indicated on the right), showing clear anomalous Hall effect in the Fe_3_GeTe_2_ device. As the temperature increases, the coercive field gradually decreases, and the hysteresis is minimal at 160 K. This trend reflects the material’s robust ferromagnetic properties at lower temperatures.

We demonstrate that our PC-assisted transfer method provides a straightforward solution for making contacts with air-sensitive magnetic 2D materials. Many magnetic 2D materials, like the very first reported CrI_3_ and CrGeTe_3_ [19,20], are particularly vulnerable to oxidation by atmospheric H_2_O or O_2_, which can significantly degrade their performance. This sensitivity poses substantial challenges in sample preparation, often rendering standard micro/nanofabrication processes incompatible. Here, we take air-sensitive ferromagnet Fe_3_GeTe_2_ as an example and demonstrate the application of our PC-assisted transfer method to make vdW contacts within a controlled environment of a glovebox. The vdW ferromagnet Fe_3_GeTe_2_ has garnered significant attention due to its high Curie temperature, strong perpendicular magnetic anisotropy, and substantial magnetostriction coefficient. In its bulk form, Fe_3_GeTe_2_ demonstrates a Curie temperature (Tc) of ~210 K [21], with its ferromagnetic properties persisting down to the monolayer limit. Although Fe_3_GeTe_2_ is metallic ferromagnet, its thin flakes are vulnerable to air, so it is difficult to use the conventional EBL process to make contacts. Our method facilitates the entire device fabrication process by simply transferring pre-fabricated Au electrodes on top of target samples inside the glovebox, maintaining the integrity of the air-sensitive material. The effectiveness of this approach is highlighted by the results of the temperature-dependent resistance (*R*–T) measurements and anomalous Hall effect (AHE) shown in Figure 3. Figure 3a depicts the *R*–T data for the few-layer Fe_3_GeTe_2_ device with PC-assisted transferred Au electrodes. A gradual increase in resistance is observed as the temperature decreases, up to the material’s Tc (~170 K). Below this temperature, the resistance decreases, reflecting the spontaneous formation of out-of-plane ferromagnetic moments and exhibiting metallic behavior. Notably, a sharp rise in resistance at approximately 30 K suggests variable-range hopping among localized carriers in the sample, consistent with a previous report [22]. This behavior reflects its intrinsic behavior and confirms the robustness of the vdW contacts fabricated via our transfer method. The *R*–T curve further supports that the bulk Tc of ~210 K decreases with reduced layer thickness, with *T*_c_ determined to be ~170 K from our measurements, as corroborated by the AHE results shown in Figure 3b. It clearly shows a nearly rectangular hysteresis loop with high signal-to-noise ratio and the coercive field gradually decreases as the temperature increases from 2 K to 180 K. These results clearly demonstrate the ferromagnetic properties of Fe_3_GeTe_2_ probed through good contact conditions. It proves that our PC-assisted transfer method effectively preserves the intrinsic properties of air-sensitive ferromagnetic 2D materials and provides excellent contacts to enable accurate and reliable measurements.

Semiconducting magnetic 2D materials, unlike the metallic magnetic 2D materials, offer additional control over their electronic states, making them highly promising for developing gate-tunable spintronic devices. This distinct capability has attracted significant attention; however, establishing effective contacts with these semiconductors poses greater challenges than with metals. Traditional methods, such as evaporated electrodes, often introduce numerous defects at the interface, resulting in localized Fermi-level pinning which limits gate-tunability. Furthermore, the evaporation process may damage the crystal lattice, altering local magnetic exchange interactions and potentially leading to magnetic domain formation at the contact. This complicates electrical measurements by making intrinsic magnetic transitions discontinuous and hinders the study of intrinsic magnetic properties.

**Figure 4:**
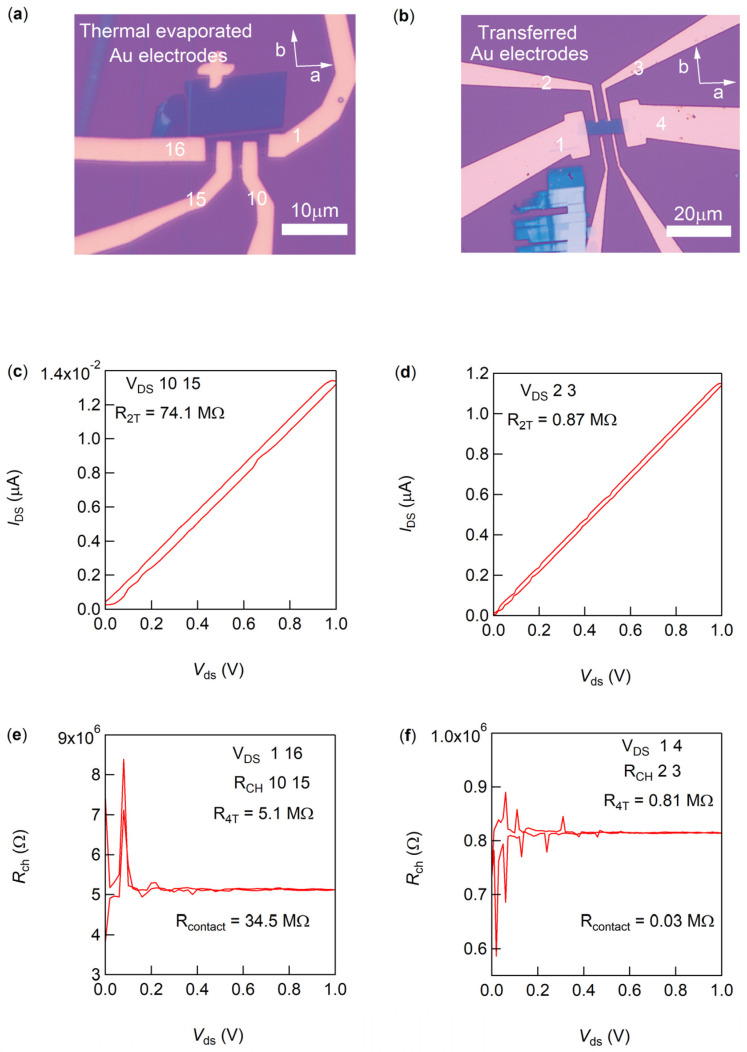
Comparison of contacts in CrSBr-based antiferromagnetic semiconductor devices. (**a**,**b**) Optical images of CrSBr devices with thermally evaporated Au electrodes (**a**) and PC-assisted transferred Au electrodes (**b**), with the crystal axes “a” and “b” marked in each image. All electrodes are numbered for easy identification of measurement configurations. (**c**,**d**) I–V curves measured using the central electrodes for the devices displayed above, from which the two-terminal contact resistance (*R*_2T_) is determined based on the slope. (**e**,**f**) Four-terminal channel resistance (*R*_4T_) for each device, calculated as *R*_4T_ = V/I. Here, a voltage (V_DS_) is applied across the outermost electrodes to measure the channel current (I), while voltage (V) is monitored at the central electrodes, which are labeled in the figure inserts.

To address these challenges, we used CrSBr, an air-stable magnetic semiconductor with a high Curie temperature [6,8,23,24], to compare contact resistance between PC-assisted transferred electrodes and thermally evaporated electrodes. Figure 4a show optical images of CrSBr samples with evaporated electrodes, while Figure 4b displays a sample with transferred electrodes. Despite both types of contacts showing similar structural integrity, significant differences were observed in performance. We used the standard four-terminal method to assess contact resistances. The two-terminal resistance between the central electrodes in this setup (Figure 4c,d) includes both the contact resistances (*R*_contact_) at each electrode and the channel resistance (*R*_channel_), expressed as *R*_2T_ = 2 × *R*_contact_ + *R*_channel_. The specific resistances and their corresponding electrodes are detailed in the figure captions. Figure 4e,f depict our use of the four-terminal method to accurately measure the channel resistance *R*_4T_ between the central electrodes, calculating *R*_contact_ as (*R*_2T_ − *R*_4T_)/2 from these values. The contact resistances for the thermally evaporated Au electrodes were significantly higher at 34.5 MΩ (Figure 4e), compared to the channel resistances of 5.1 MΩ, respectively. In stark contrast, the transferred electrode contact resistance was dramatically lower at 0.03 MΩ (Figure 4f), with a channel resistance of 0.81 MΩ, showcasing a reduction of about four orders of magnitude compared to the evaporated electrodes. This substantial reduction underscores the superior contact quality achieved with PC-transferred electrodes, making them highly suitable for applications in magnetic semiconductors.

**Figure 5:**
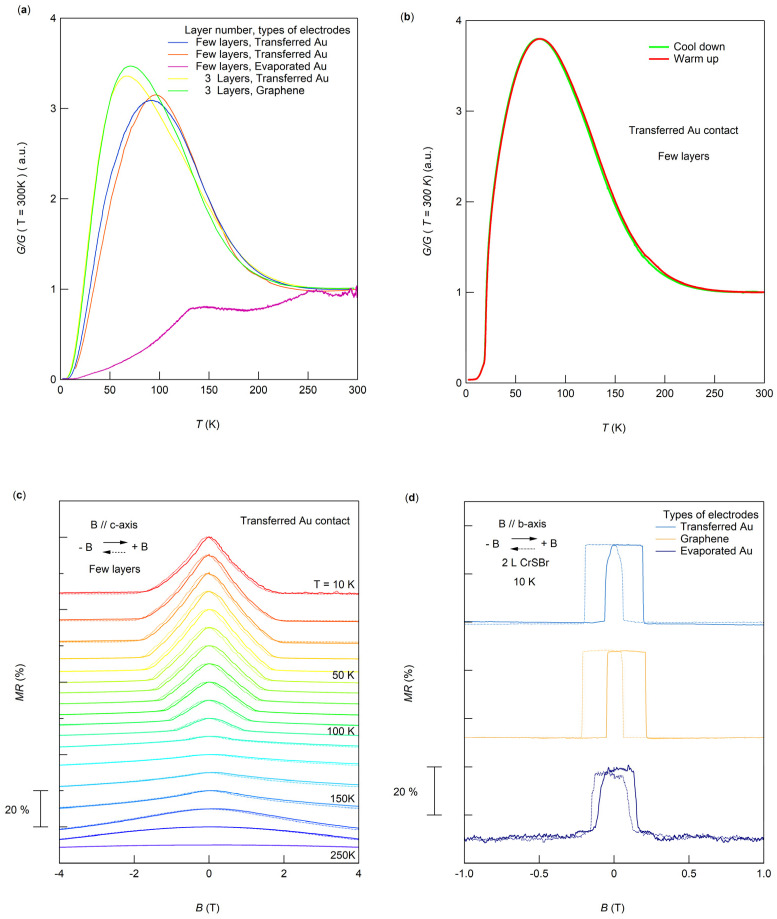
Transport properties of the antiferromagnetic semiconductor CrSBr measured with different types of contacts. (**a**) Normalized conductance (defined as G/G (T = 300 K)) as a function of temperature (T) measured for CrSBr devices with various thicknesses and types of contacts. (**b**) Comparison of normalized conductance-vs.-temperature curve for the initial cooling-down process with the curve for the warming-up process after prolonged low-temperature measurements for a few-layer CrSBr device with PC-assisted transferred electrodes. (**c**) Magnetoresistance ratio, defined as MR (%) = (*R*(B) − *R*(0T))/*R*(0T) measured at various temperatures for the same few-layer CrSBr devices in (**b**). The external magnetic field applied along the c-axis (perpendicular to the a,b-plane). (**d**) Magnetoresistance ratio measured at 10 K for the bilayer CrSBr devices with PC-assisted transferred Au electrodes, graphene electrodes, and thermally evaporated Au electrodes. The external magnetic field applied along the easy axis (b-axis of CrSBr).

Further, we performed two-terminal measurements to detect the intrinsic magnetic properties of CrSBr with different types of contacts. Figure 5a shows the normalized conductance-versus-temperature curves for CrSBr, with sample thickness and contact types indicated in the insert. Unlike conventional semiconductors where resistance typically decreases as the temperature drops, our magnetic semiconductor devices exhibited an initial increase in resistance, which then decreased as the temperature approaches the Néel temperature due to reduced spin scattering with the formation of magnetization [25]. The maximum conductivity relative to that at 300 K, G_max_/G (300 K), reflects the magnetic signal’s contribution to total conductivity. As shown in Figure 5a, the much larger value of G_max_/G (300 K) for the thin-layer sample with transferred electrodes compared to that with thermally evaporated electrodes strongly indicates that the transferred electrodes have better preservation of intrinsic magnetic signals and improved contact quality. The *R*–T curves align well with previous reports for CrSBr [24,25], confirming the reliability of data measured from transferred electrodes. With samples of similar thickness, the *R*–T curves for graphene contacts and transferred electrodes closely resemble each other, suggesting that transferred electrodes can achieve nearly van der Waals-type interfaces similar to graphene.

Figure 5b presents the normalized conductance-versus-temperature curves for a few-layer CrSBr sample with PC-transferred Au electrodes during both cooling and warming cycles. We performed extensive low-temperature measurements after the cooling-down process and measured the magnetoresistance (MR) with the magnetic field oriented along the c-axis (perpendicular to the a,b-plane), as shown in Figure 5c. These smooth MR results clearly show the gradual decrease in the coercive field as the temperature increases, consistent with a previous report [25]. The conductance–temperature curve upon warming in Figure 5b was taken after multiple cooling-down processes and prolonged measurements. It aligns perfectly with the initial cooling curve, proving the robustness and reliability of the sample with the transferred electrodes over extended experimental durations.

Additionally, we measured the MR at 10K for bilayer CrSBr samples with different contact conditions, where MR (%) = (*R*(B) − *R*(0T))/*R*(0T). The layer number was identified by optical contrast following the same method used in a previous report [25]. Figure 5d displays the MR behavior under various contact methods. Without an external magnetic field, CrSBr exhibits an A-type antiferromagnetic structure, characterized by intralayer ferromagnetic and interlayer antiferromagnetic ordering [25,26]. Upon applying a magnetic field along the easy axis, a sharp drop in MR is observed at 0.2T due to spin flip, which overcomes anisotropy barriers to form an interlayer ferromagnetic state, reducing resistance by decreasing interlayer spin-scattering. This transition field is consistent with previous studies [8,27] for bilayer CrSBr. It should be noted that we observed a large hysteresis in MR at 10 K, similar to that observed by PL measurements in the bilayer CrSBr sample [28]. This hysteresis may originate from substrate-doping, which influences the energy required to transition between antiferromagnetic and ferromagnetic states. Comparing MR signals from graphene and transferred electrodes shows similar intensity and sharpness, while evaporated Au electrodes produce a less distinct signal, likely due to sample damage during evaporation that introduces local magnetic domains, distorting the intrinsic signal. This comparison further confirms the reliability of PC-transferred electrodes for exploring intrinsic properties and device applications of magnetic semiconductors.

## 4. Discussion

Our results demonstrate excellent van der Waals contact with magnetic 2D materials, including air-sensitive Fe_3_GeTe_2_ and semiconducting CrSBr, showcasing the versatility and effectiveness of this method in enhancing magnetic transport and device performance. In fact, our method has the potential to transfer both electrodes and 2D materials simultaneously, as PC itself is compatible with standard dry-transfer techniques [18], facilitating the fabrication of more complex magnetic heterojunction devices. Looking ahead, there is potential for transfer magnetic electrodes such as Ni and Co, which would significantly accelerate the fabrication of high-performance, complex spin-transport devices and expand the application of magnetic 2D materials. Additionally, our method shows promise for fabricating wafer-scale array devices, although challenges such as air bubbles between large-area PC and PDMS stamp and the tendency of PC to form wrinkles during the flipping process need to be addressed.

We can compare the advantages of the PC-assisted electrode transfer method with previously reported mechanical transfer approaches. Firstly, the PC-assisted method we developed eliminates the need for complex pre-treatment steps. For instance, PMMA transfer requires HMDS functionalized on the sacrificial substrate [13,14,16,17] to achieve weak adhesion or prior growth of monolayer graphene [15]. These cumbersome processes undoubtedly increase both time and cost. Secondly, our technique can be carried out in a glovebox environment, making it ideal for air-sensitive 2D materials. Previous transfer methods with high success rates often involved introducing water during the electrode separation process, such as using water-soluble polymers like polyvinyl alcohol (PVA) [15] or quasi-van der Waals epitaxy of metals on fluorophlogopite mica (F-mica) [29], which is not suitable for air-sensitive 2D materials, particularly magnetic materials. Therefore, our method represents a more universal, modification-free electrode transfer approach, greatly optimizing both process complexity and time cost. With a success rate of nearly 100% in separating Au electrodes on Si substrates so far, our method holds potential for integration with other defect-free techniques [30,31] to broaden its applicability and improve performance. This could make it an indispensable tool in advancing electrode technology for a variety of applications.

## 5. Conclusions

We have introduced a universal, low-cost, and efficient technique for achieving van der Waals contacts with a variety of 2D materials without the need for an additional lithography process. This PC-assisted transfer method has demonstrated superior interface contact quality and effective preservation of intrinsic magnetism in magnetic 2D materials such as air-sensitive Fe_3_GeTe_2_ and semiconducting CrSBr. This approach is thus ideal for exploring intrinsic properties and device applications of magnetic 2D materials. Moreover, our transfer technique is compatible with standard dry-transfer techniques and is scalable to large wafer sizes, offering significant potential to advance the exploration of magnetic 2D materials and their integration into spintronic applications.

## Data Availability

The data are available from the corresponding authors upon reasonable request.

## Supplementary Materials

The following supporting information can be downloaded at https://www.mdpi.com/article/10.3390/mi15111401/s1: Video S1: PC film transfer and flipping onto PDMS handling.
